# New Polymer Inclusion Membrane Containing β-Cyclodextrin Polymer: Application for Pharmaceutical Pollutant Removal from Waste Water

**DOI:** 10.3390/ijerph16030414

**Published:** 2019-01-31

**Authors:** Lamia Moulahcene, Mohamed Skiba, Frederic Bounoure, Mohamed Benamor, Nicolas Milon, Francois Hallouard, Malika Lahiani-Skiba

**Affiliations:** 1 UNIROUEN, DC2N INSERM U1239-Galenic Pharmaceutical Team, UFR of Health, Normandy University, 22 Bd Gambetta, FR-76000 Rouen, France; lamia19851919@hotmail.fr (L.M.); frederic.bounoure@univ-rouen.fr (F.B.); nicolas.milon@univ-rouen.fr (N.M.); francois.hallouard@gmail.com (F.H.); 2 Laboratory of membrane processes and of separation and recovery techniques, Faculty of Technology, Abderrahmane-Mira University, Route de Targua Ouzemmour, DZ-06000 Bejaia, Algeria; mohamedbenamor@yahoo.fr; 3 Service de Pharmacie, Hospices Civils de Lyon, Chemin du Grand-Revoyet, FR-69395 Pierre-Bénite, France

**Keywords:** cyclodextrin polymer, polymer inclusion membrane (PIM), pharmaceutical pollutant, ibuprofen, progesterone, wastewater

## Abstract

We present herein the preparation of novel polymer inclusion membranes (PIMs) containing insoluble β-CD polymer as a carrier, polyvinyl chloride as a base polymer, and dibuthylphtalate (DBP) as a plasticizer in varying proportions. The prepared PIMs can be obtained by a simple, fast, and high-yield preparation process. Physicochemical characterizations of such membranes occurred in a homogeneous structure. In addition, Fourier-transform infrared Spectroscopy (FT-IR) analysis found that DBP was inserted between these polymeric chains by non-covalent interactions. This led to a spacing of PVC/poly(β-cyclodextrin) chains inducing a better access of guest molecules to PIM cyclodextrins. To achieve the elimination of ibuprofen and progesterone, two examples of emerging environmental contaminants that can lead to possible alterations to aquatic environments and affect human health, the effect of three operating parameters was studied (pH, the proportion of β-cyclodextrin polymer, and wastewater agitation). The proportion of β-cyclodextrin polymer and wastewater agitation had a favorable influence on drug extraction at 10 ppm. The PIMs containing β-cyclodextrin polymer was unstable in basic conditions and was more effective at acidic pH. These initial results demonstrate the high potential for drug extraction of this polymer.

## 1. Introduction

Several methods have been used for drug extraction from environmental wastewater, such as photodegradation [1], nanofiltration and ultrafiltration [2,3], ozone oxidation [4], electrodialysis membrane [5], and coagulation-floculation and flotation [6]. Most of these techniques suffered either from technical or economic problems, were associated with a long processing time, high energy consumption or required a large quantity of hazardous materials used, while the others were not sufficiently selective or cost-efficient in the case of dilute solutions. 

However, polymer inclusion membrane (PIM) is an attractive method for water treatment because of its high efficacy in case of dilute solutions, ease of handling, and selectivity. According to Almeida et al., PIMs are a type of liquid membrane composed of a liquid phase and a base polymer. The base polymer such as poly(vinyl chloride) (PVC) is the skeleton of the membrane providing mechanical strength. The liquid phase is mainly an extractant/carrier which is responsible for binding the pollutant by complexation or ion-pair formation [7,8]; one such carrier is cyclodextrins (CDs). 

CDs are cyclic oligosaccharides consisting of 6 or more glucopyranose units. Due to their structural features, they have an external hydrophilic surface and a hydrophobic cavity in which a wide variety of guest molecules may form inclusion complexes [9]. Native or polymerized CDs are widely used as a carrier in affinity membranes [10,11,12] for the removal of contaminants such as xylene [13], heavy metals [14], and hydroquinone [15].

Insoluble poly(cyclodextrin) polymers can be obtained by condensation of CDs with a bi- or poly-functional substance such as epichlorohydrin [16], hexamethylene diisocyanate (HMDI) [17], and citric acid [18,19]. Poly(cyclodextrin) polymers are used in the removing of pollutants from wastewater such as dyes [20,21], phenol [16], and aromatic amines [17] due to the ability of CDs to form inclusion complexes. 

Recently pharmaceutical substances have received growing attention as emerging environmental contaminants that can affect aquatic environments and human health [22,23]. Progesterone, a natural hormone used as a drug to control the reproductive function and for postmenopausal therapy, and ibuprofen, a non-steroidal anti-inflammatory drug that is among the most commonly consumed drug worldwide, were frequently detected in surface water [24], rivers and lakes [25,26,27,28], hospital and municipal water [29,30], and in some cases in drinking [31,32] and agricultural water [27,33].

In the present study we present the preparation of novel PIMs containing insoluble β-CD polymer as a carrier, polyvinyl chloride as a base polymer, and dibuthylphtalate (DBP) as a plasticizer in varying proportions; these membranes were analyzed using Fourier-transform infrared spectroscopy (FT-IR), thermogravemetric analysis, and scanning electron microscopy (SEM) analysis. Afterwards, these membranes were used to remove two pharmaceutical substances, and finally stability was also studied. The novelty of the present work is the preparation of a composite selective membrane which can remove micropollutants at low concentrations.

## 2. Materials and Methods

### 2.1. Materials

**Figure 1:**
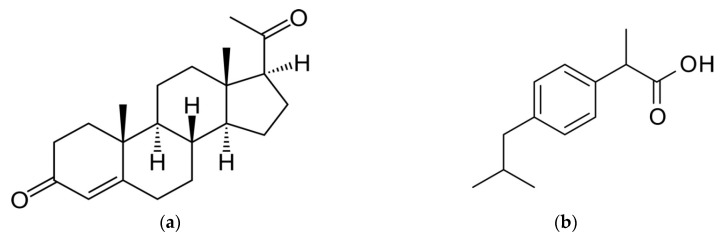
Molecular structures of progesterone (**a**) and ibuprofen (**b**).

Ibuprofen and progesterone (purity ≥ 99.0%) were purchased respectively from Hubei Biocause Pharmaceutical Coprporation Jingmen, Hubei, China) and Upjohn Company (Kalamazoo, MI, USA; Figure 1). They were used without further purification. Native β-CDs were obtained from Roquette (Lestrem, France). Polyvinyl chloride (PVC) with a molecular weight of 230,000 Da and dibutylphtalate (DBP) were purchased respectively from Sigma-Aldrich (Saint-Louis, MI, USA) and VWR^TM^ (Radnor, PA, USA). All other reagents were of analytical grade.

### 2.2. Synthesis of the Insoluble Β-Cyclodextrins Polymer

Poly(β-cyclodextrin) polymer was synthesized by a direct melt copolycondensation process according to the method reported by Skiba and Lahiani-Skiba [18]. Briefly, a mixture of a known amount (w/w) of β-cyclodextrins, citric acid, and sodium phosphate dibasic was transferred into a reactor which was maintained at temperature between 140 and 150 °C for a fixed period of time. The obtained solid form was dissolved in water and dialyzed using a polyether sulfate membrane filter with a molecular weight cut off of 10,000 Da. After dialysis, the resulting solution was spray dried using a Mini Sprayer Dryer B-290^®^ (BÜCHI, Flawil, Switzerland). Two polymer fractions were obtained: a soluble and insoluble one. The insoluble polymer was washed with methanol and dried at 60 °C [9,19], the insoluble one is used herein to prepare a polymer inclusion membrane.

### 2.3. Preparation of the Polymer Inclusion Membrane

The appropriate amount of PVC (0.2 g) was dissolved in 10 mL of tetrahydofuran (THF). A separate suspension containing insoluble poly(β-cyclodextrin) polymer and/or DBP was prepared. These were mixed together and stirred for 30 min to form a final suspension. This suspension was then spread on a 9 cm-diameter flat-bottom glass Petri dish, which was covered with a glass plate in such a way that aeration was possible in order to allow the total evacuation of the solvent, which takes more than 24 h. After evaporation of THF, the composite membrane was peeled off the Petri dish.

The weight of the P-β-CD used for the membrane preparation varied as follows: 20 wt%, 40 wt%, 50 wt% and 60 wt%. 

### 2.4. Membrane Characterization

#### 2.4.1. Fourier Transform Infrared Spectroscopy

Attenuated Total Reflection (ATR)—Fourier transform-infrared spectroscopy (FT-IR) measurements were performed on PIMs. Samples were placed on a wedged (Ge-ATR crystal), pressed with a force of 80 N and spectra were then recorded using a Spectrum One^®^ (Perkin-Elmer, Waltham, MA, USA). A freshly cleaned crystal was used as a reference. Each analysis was conducted between 4000 cm^−1^ and 400 cm^−1^, at a resolution of 4 cm^−1^, and with 20 scans. All measurements were performed in triplicate.

#### 2.4.2. Scanning Microscopy Analysis

A concentrated aqueous dispersion of cyclodextrin-based polymer was finely spread over a slab and dried under vacuum. The sample was shadowed in a cathodic evaporator with a gold layer (20 nm thick). The surface morphology and the section of the membranes were observed by SEM using a Cambridge S360^®^ (Leica, Solms, Germany) and the large field detector (LFD) mode.

#### 2.4.3. Thermogravemetric Analysis

Mass losses were recorded with TGA 4000^®^ (Perkin-Elmer, Waltham, MA, USA) on 5 mg samples in open pans at a heating rate of 10 °C/min in the 30 °C–600 °C temperature range under a nitrogen gas flow (25 mL/min). All measurements were performed in triplicate.

### 2.5. Application of Membrane to Pharmaceuticals Removal

**Figure 2:**
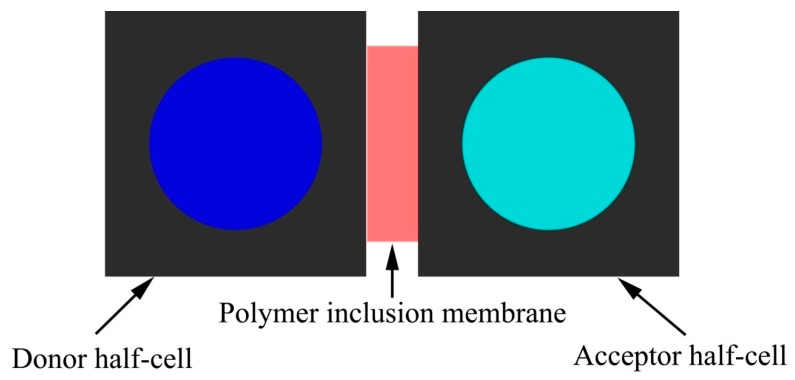
Experimental setup for the pharmaceuticals extraction with Polyvinyl chloride/dibutylphtalate/poly(β-cyclodextrin) membrane extraction cell. In the donor half-cell there is initially 60 mL of aqueous solution of the drug (ibuprofen or progesterone) at 10 ppm. Sample for measurements were taken from acceptor half-cell.

As illustrated in Figure 2, adsorption experiments were performed by using a permeation cell that consists of two identical cylindered compartments (half-cell volume of 60 mL). Stock solution of ibuprofen (20 mg/L) was prepared in deionized water. For progesterone, stock solution was made with a mixture of deionized water/ethanol (60/40 (v/v)) at a drug concentration of 20 mg/L. The experimental solutions with desired concentration (10 ppm of each drug) were obtained by successive dilution of these stock solutions with deionized water.

A defined volume (60 mL) of ibuprofen or progesterone solution was transferred to the donor half-cell under agitation. The acceptor cell is also 60 mL. The time-courses of the ibuprofen and progesterone uptake over 6 h were followed (to obtain the sorption equilibrium) by determination of the concentration of ibuprofen and progesterone in the acceptor half-cell under agitation. Drug content (ibuprofen or progesterone) in wastewater was determined using a UV/VIS-spectrophotometer (JASCO, V-R30, Lisses, France) at 223 or 250 nm (corresponding respectively to a maximum absorbency of progesterone and ibuprofen). The sample volume was 800 µL of wastewater. Calibration curve of ibuprofen was prepared by measuring absorbance of samples with predetermine concentrations. The removal experiments were conducted at 20 °C, varying pH (1.0, 7.3 and 10.0), the proportion of β-cyclodextrin polymer (20% to 60%), and wastewater agitation (400 to 600 rpm). Each measurement was made in triplicate. 

## 3. Results and Discussion

### 3.1. Characterization of Polymer Inclusion Membranes

#### 3.1.1. Scanning Electronic Microscopy

**Figure 3:**
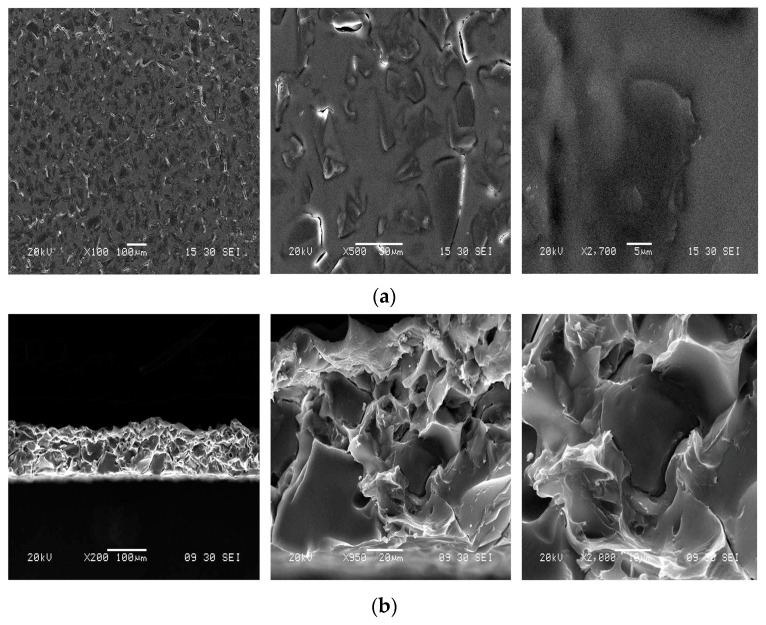
SEM acquisition at different magnifications of the surface (**a**) and the cross-section (**b**) of the PVC/DBP/poly(β-cyclodextrin) membrane.

SEM images of the surface and cross-section of the PVC/DBP/poly(β-cyclodextrin) membrane are presented in the Figure 3. For this PIM, a homogenous incorporation of poly(β-cyclodextrin) polymer was found at different magnifications used, limiting potential heterogeneous behavior of the membrane. The PVC/DBP/poly(β-cyclodextrin) membrane seemed to present a poor porosity; this was the consequence of the slow THF evaporation process during the PIM preparation, leading to the formation of very small pores that were less than few µm and were thus invisible on SEM acquisitions. 

#### 3.1.2. Thermogravimetric Analysis

**Figure 4:**
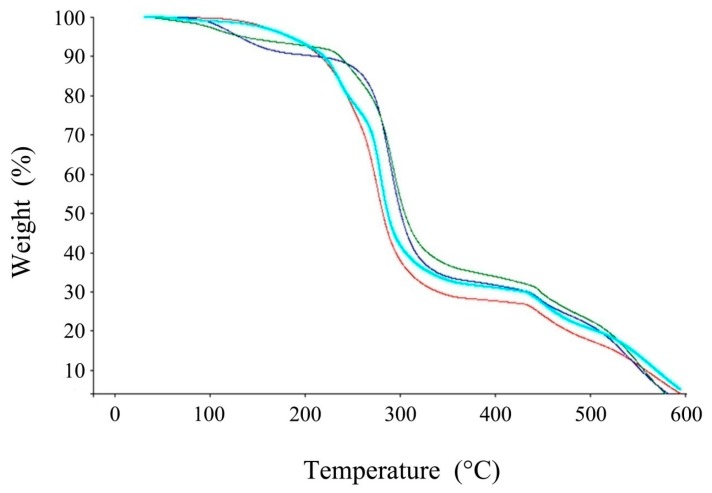
TGA profiles of PVC (dark blue) and different polymer inclusion membranes: PVC/DBP (red), PVC/poly(β-cyclodextrin) (green) and PVC/DBP/poly(β-cyclodextrin) (light blue). Each experiment was performed in triplicate.

The thermal stability of the PIMs and their constituents was checked by thermogravimetric analysis (TGA) (Figure 4). TGA results showed a dehydration (mass loss at 100 °C) followed by two steps of degradation. The first degradation step was between 250 °C and 300 °C and is attributed to the degradation of the principal chain of the polymer; the second step at 450 °C indicated PVC carbonization. The PVC/ poly(β-cyclodextrin) membrane presented a TGA profile similar to PVC; a dehydration step followed by two degradation steps, respectively between 200 °C and 280 °C and at 450 °C. This showed the predominant influence of PVC on the PVC/poly(β-cyclodextrin) membrane. Conversely, the addition of DBP to PVC or to PVC/poly(β-cyclodextrin) led to a progressive alteration of PIM starting at 200 °C instead of 250 °C and without a dehydration step showing a significant modification of the PIM TGA profile. The significant influence of DBP, a plasticizer, is probably due to its incorporation between the PVC or PVC/poly(β-cyclodextrin) chains. 

#### 3.1.3. FT-IR Spectroscopy

**Figure 5:**
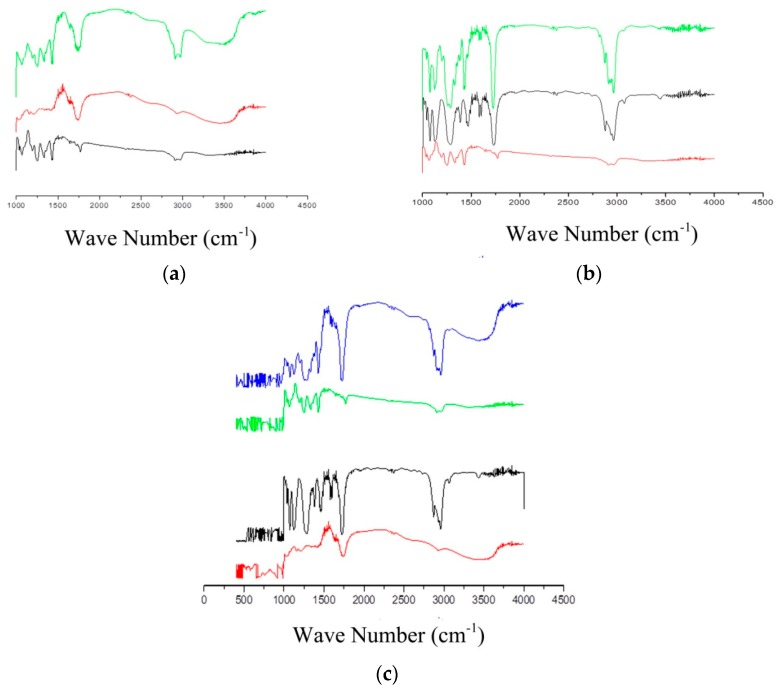
FT-IR spectra of different PIMs and their constituents. (**a**) PVC/poly(β-cyclodextrin) membrane (green), PVC (black) and poly(β-cyclodextrin) (red). (**b**) PVC/DBP membrane (green), PVC (red) and DBP (black). (**c**) PVC/DBT/poly(β-cyclodextrin) membrane (blue), PVC (green), DBP (black) and poly(β-cyclodextrin) (red). Each measurement was made in triplicate.

FT-IR analysis provided information regarding the nature of the chemical interactions between different components of the PIMs. Figure 5 summarizes the FT-IR of different PIMs (PVC/DBP, PVC/poly(β-cyclodextrin) and PVC/DBP/poly(β-cyclodextrin)) and their constituents (PVC, DBP, and poly(β-cyclodextrin)). 

The peaks between 2850–2956 cm^−1^ which are attributed to –CH_3_ group in DBP and the peak between 2986–2980 cm^−1^ attributed to –CH in PVC, are shifted to 2850–2956 cm^−1^ in the PVC/DBP/poly(β-cyclodextrin) and PVC/DBP membranes. This may be explained by the overlapping of the PVC and DBP spectra. The shift of the C=O peak in the ester group (O=C-O-R) from 1746 cm^−1^ in native poly(β-cyclodextrin) to 1721 cm^−1^ in the PVC/DBP/poly(β-cyclodextrin) membrane is due to the formation of hydrogen bonds between poly(β-cyclodextrin) and DBP. In the spectra of the PVC/poly(β-cyclodextrin) membrane, a shift of the peaks attributed to the H-C-H in the PVC from 2986–2980 cm^−1^ to 2869–2963 cm^−1^ and the C=O in the ester group of the P-β-CD from 1746 cm^−1^ to 1740 cm^−1^ are also of note. This is attributed to the formation of hydrogen bonds with these functional groups. All of these results confirmed the expected interactions between PVC, DBP, and poly(β-cyclodextrin) which were non-covalent interactions between DBP, the plasticizer, and PVC or poly(β-cyclodextrin).

**Figure 6:**
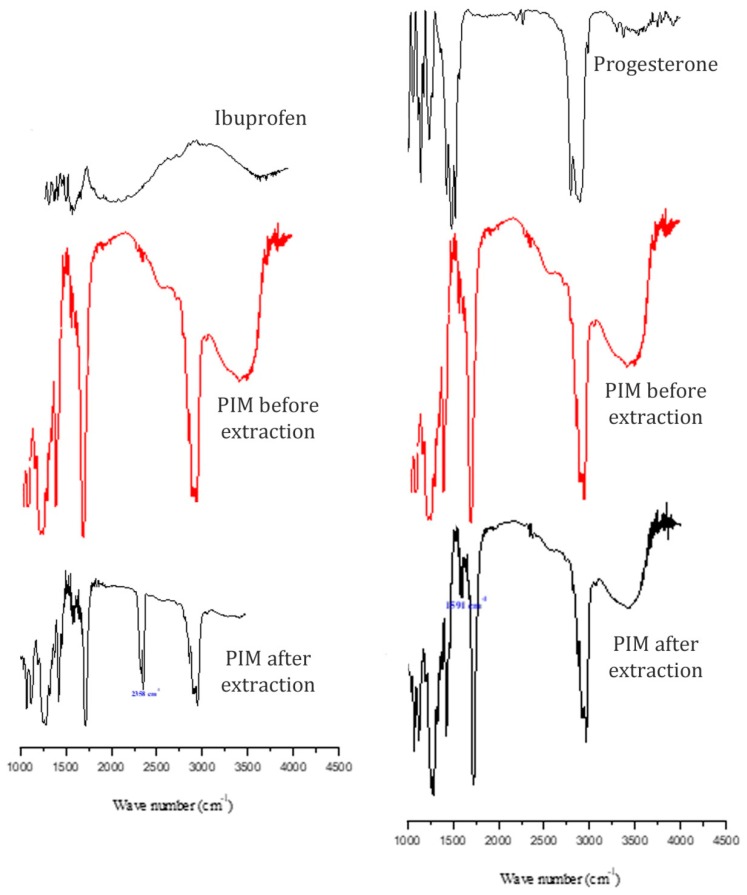
FT-IR spectra of different PIMs nefore and after extraction of Ibuprofen and progesterone.

To understand how drugs will be removed by these PIMs, a comparison of the infrared spectra of progesterone, ibuprofen, and membranes after extraction of pharmaceuticals are shown in Figure 6. A significant change in PIM spectra after pharmaceutical extraction was observed only for progesterone. For the latter, the peak at 1591 cm^−1^ attributed to the stretching of C=C in progesterone which proves the extraction of the progesterone by this membrane. After ibuprofen extraction, we determined that a new peak at 2358 cm^−1^ appeared in the PIM spectrum, indicating the presence of ibuprofen in the membrane. 

#### 3.1.4. Polymer Inclusion Membranes Stability Study

Membrane stability, particularly against both fluid agitation and pH, is also a critical factor for decontamination application by wastewater filtrations. Indeed, an alteration of membrane integrity can make inefficient the filtration.

Concerning the influence of agitation, this investigation was performed on the PVC/DBP/poly(β-cycodextrin) membrane. Its stability was expressed by the weight loss calculated according to the following Equation (1):

(1) $$\mathrm{Weight}\;\mathrm{loss}=\frac{m_{i}-m_{f}}{m_{f}}\times 100\%$$

where *m_i_* and *m_f_* are respectively the membrane weight after and before a membrane agitation during 6 h.

Agitation did not have an effect on the membrane stability (Table 1). Nevertheless, it is notable that the weight loss increased with the agitation speed; from 3% at 400 rpm to 6.8% at 500 rpm; this did not notably change at 600 rpm. The observed weight loss is attributed to the elimination of the β-cyclodextrin polymer remaining on the PIM surface. This membrane could therefore be considered to be stable.

Concerning the influence of pH on PIM stability, this investigation was performed in an acidic condition using a solution of HCl 0.1 M (pH = 1.0), in neutral conditions using phosphate buffer (pH = 7.3), and in a basic condition using a solution of NaOH 0.1 M (pH = 10.0). For the PVC/DBP membrane, pH had no influence on stability; weight loss did not exceed 1% in all cases (Table 2). As expected, PVC/poly(β-cyclodextrin) and PVC/DBP/poly(β-cyclodextrin) membranes were altered at high pH and were stable in acid and neutral media. Basic aqueous solutions are optimal conditions for the hydrolysis of ester functions present in poly(β-cyclodextrin) between β-cyclodextrins and citric acid used as spacer. DBP as the plasticizer was not covalently bound to PVC or β-cyclodextrins in PIM but was only inserted between the PVC/poly(β-cyclodextrin) polymeric chains, which explains the absence of pH influence on PVC/DBP membrane. These results demonstrated the crucial role of the acidic and ester groups in the β-cyclodextrin polymer in the integrity of poly(β-cyclodextrin) based membranes and thus their drug removal efficiency. 

### 3.2. Removal of Pharmaceuticals and Effect of Operating Parameters

The effect of different process factors was tested for ibuprofen and progesterone, such as the choice of PIM, the proportion of β-cyclodextrin in the membrane, and initial pH. The initial concentration in the donor half-cell was 10 ppm for both drugs (progesterone and ibuprofen). 

#### 3.2.1. Effect of the Type and Composition of The Membrane for Drug Extraction

**Figure 7:**
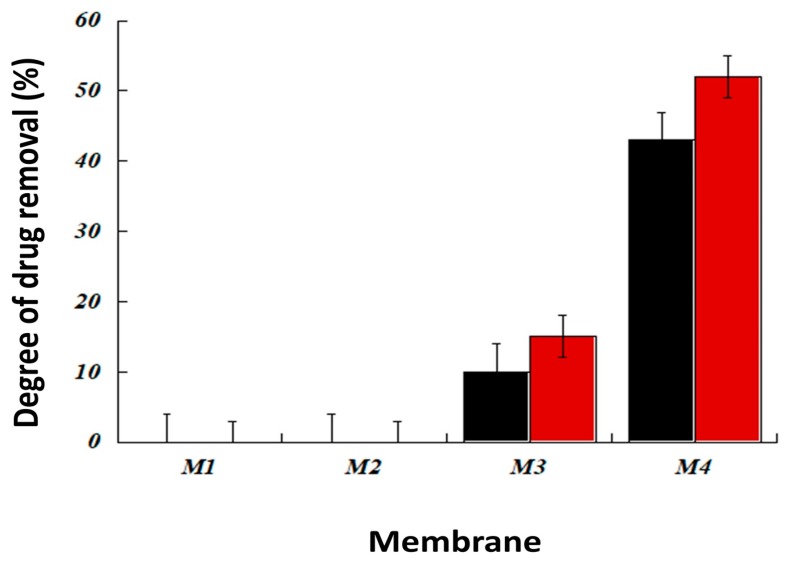
Influence of membrane composition on extraction of residual drug in wastewater. Different membranes were tested: PVC (M1), PVC/DBP (M2), PVC/poly(β-cyclodextrin) (M3) and PVC/DBP/poly(β-cyclodextrin) (M4). Two drugs were tested at an initial concentration of 10 ppm: ibuprofen (red circles) and progesterone (black squares). The extraction process was performed over 6 h. Each essay was performed in triplicate.

**Figure 8:**
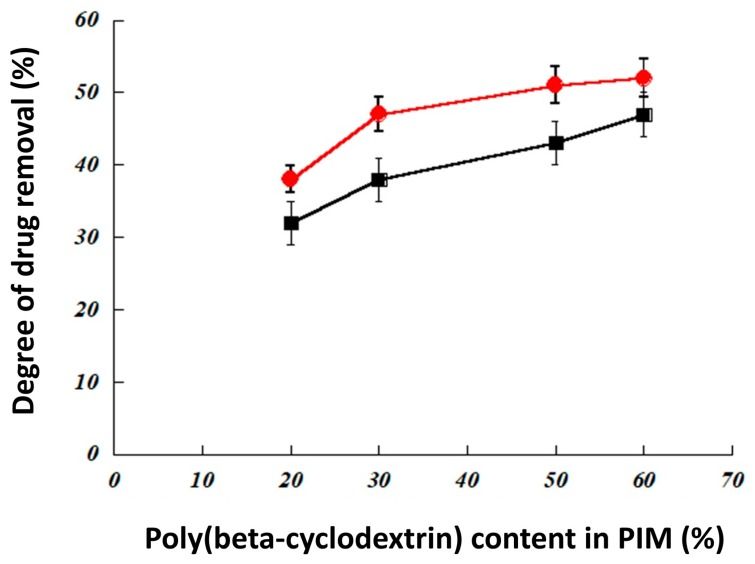
Influence of poly(β-cyclodextrin) content in the PVC/DBP/poly(β-cyclodextrin) membrane on the extraction of residual drug in wastewater. Two drugs were tested at an initial concentration of 10 ppm: ibuprofen (red circles) and progesterone (black squares). The extraction process was performed over 6 h. Each essay was performed in triplicate.

Figure 7 summarizes the efficiency of different membranes for the extraction of both ibuprofen and progesterone. As expected, poly(β-cyclodextrin) polymer was required in the membrane to obtain drug-extraction properties. Indeed, poly(β-cyclodextrin) polymer is the only constituent composed of an inclusion agent (β-cyclodextrin) previously described to form complexes efficiently with both ibuprofen and progesterone [34,35]. This hypothesis was confirmed by the study of the influence of poly(β-cyclodextrin) polymer content in PIM (Figure 8); the drug extraction efficiency of PIM increased with this polymer content until 50 wt% above which it became nearly constant for the two molecules. 

In addition, DBP had a favorable influence on PIM for drug extraction (Figure 7). This could be the consequence of DBP insertion between PVC/poly(β-cyclodextrin) polymeric chains by non-covalent interactions which leads to a spacing of PVC/poly(β-cyclodextrin) chains inducing better access of guest molecules to PIM cyclodextrins. 

#### 3.2.2. Effect of pH

**Figure 9:**
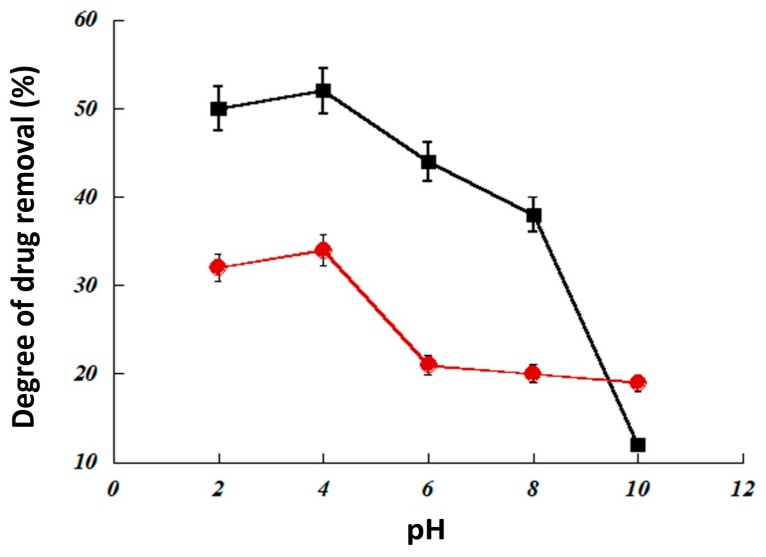
Influence of pH on extraction of residual drug in wastewater. Two drugs were tested at an initial concentration of 10 ppm: ibuprofen (red circles) and progesterone (black squares). The extraction process was performed over 6 h. Each essay was performed in triplicate.

The pH has a significant effect on the integrity of our PIMs, particularly in basic conditions as demonstrated previously. However, pH could also have an influence on the drug extraction efficiency of the membranes. As expected, the drug extraction of the PIMs was influenced by the pH; drug extraction decreased as a function of increasing medium pH (Figure 9), which may be explained by hydrolysis of the poly(β-cyclodextrin) in PVC/DBP/poly(β-cyclodextrin) membrane in basic conditions. These results demonstrate the necessity to respect the pH condition during pollutant extraction by such membranes. 

## 4. Conclusions

In the present work we have succeeded in preparing novel PIMs using a simple, fast, and high-yielding preparation process. Physicochemical characterizations of such membranes showed a homogeneous structure and high thermal stability. In addition, DBP was inserted between these polymeric chains through non-covalent interactions. This led to a spacing of PVC/poly(β-cyclodextrin) chains, thereby inducing better access of guest molecules to PIM cyclodextrins. The obtained PIM was stable under high speed agitation (600 rpm) and in acidic and neutral media, but is not stable in an alkaline medium. 

To achieve the elimination of ibuprofen and progesterone, the effect of three operating parameters was studied (pH, the proportion of β-cyclodextrin polymer, and wastewater agitation). The proportion of β-cyclodextrin polymer and wastewater agitation had a favorable influence on drug extraction at 10 ppm. The PIMs containing β-cyclodextrin polymer was unstable in basic conditions and was more effective at acidic pH. These initial results demonstrate the high potential for drug extraction of this polymer. The next step will be the study of the efficacy of this polymer to extract these molecules in real samples. 

## Figures and Tables

**Table 1:** Influence of wastewater agitation on the stability of PVC/DBP/poly(β-cyclodextrin) membrane.

Agitation (rpm)	400	500	600
Weight loss (%)	3.0	6.7	6.9

**Table 2:** Influence of wastewater pH on the stability of different polymer inclusion membranes: PVC/poly(β-cyclodextrin) membrane; PVC/DBP membrane PVC/DBP/poly(β-cyclodextrin) membrane in different aqueous solutions (HCl at 0.1 M, NaOH at 0.1 M and phosphate buffer (pH = 7.3)).

Membrane	PVC/poly(β-cyclodextrin)			PVC/DBP			PVC/DBP/poly(β-cyclodextrin)		
Medium	HCl at 0.1 M	Phosphate buffer	NaOH at 0.1 M	HCl at 0.1 M	Phosphate buffer	NaOH at 0.1 M	HCl at 0.1 M	Phosphate buffer	NaOH at 0.1 M
Weight loss (%)	3.9	4.5	17	0.5	0.5	0.5	4	4	9
